# A Case of Resistance to Thyroid Hormone with Chronic Thyroiditis: Discovery of a Novel Mutation (I54V)

**DOI:** 10.1155/2011/584930

**Published:** 2011-10-09

**Authors:** I. Kammoun, C. Bouzid, H. Kandara, L. Ben Salem, Z. Turki, C. Ben Slama

**Affiliations:** Endocrinology and Diabetology Department, National Nutrition Institute, 1007 Tunis, Tunisia

## Abstract

Resistance to thyroid hormone (RTH) is a rare disorder characterized by variable tissue hyporesponsiveness to thyroid hormone, usually caused by mutations in the thyroid hormone receptor beta (TR**β**). It has been reported that the serum of patients with RTH is free of auto-antibodies against thyroglobulin (Tg) and thyroid peroxidase (TPO), except in rare cases where coincidental autoimmune thyroiditis is also present. We describe a 13-year-old girl with chronic thyroiditis and RTH. This patient had increased plasma free T3, free T4 at the upper limits with unsuppressed TSH. She had peripheral manifestations of thyroid hormone excess, hypertension and growth acceleration. Anti-TPO antibodies were positive. Sequence analysis of the TR**β** gene was performed and revealed a novel mutation I54V in exon 4. The same mutation was also found in the mother and two asymptomatic sisters. The clinical presentation of our patient is not habitual in RTH because growth retardation is frequently reported in this syndrome. The association between RTH and thyroiditis complicate the management of the hypothyroidism.

## 1. Introduction

Thyroid hormone resistance (RTH) is a rare and usually autosomal dominant disorder characterized by reduced target tissues responsiveness to thyroid hormones. Since Refetoff et al. described this syndrome in 1967 [1], over 1000 cases have been identified [2].

Thyroid hormone secretion is stimulated by thyroid-stimulating hormone (TSH), itself under a negative feedback by thyroid hormones. The RTH is characterized by high levels of circulating thyroid hormone and inappropriately normal or elevated value of TSH.

Formerly, RTH was subdivided into generalized RTH (GRTH) and pituitary RTH (PRTH) [3]. PRTH is extremely rare and, in general, its diagnosis is made largely on the basis of the presence of the clinical signs and symptoms of hyperthyroidism [4]. Based on symptoms and signs, this subclassification does not have a logical basis because these two presentations are encountered in individuals with the same mutation [5]. 

The linkage between RTH and the TR*β* gene was found in 1988 [6]. Since then, approximately 100 mutations have been detected in this gene [7, 8]. It has been reported that the serum of patients with RTH is free of autoantibodies against thyroglobulin (Tg) and thyroid peroxidase (TPO), except in the rare instances where coincidental autoimmune thyroiditis is also present [9].

In this study, we describe a 13-year-old girl with chronic thyroiditis and RTH. She had manifestations of peripheral thyroid hormone excess, hypertension, growth acceleration, inappropriate secretion of TSH, and increased anti-TPO antibodies. Genetic analysis revealed a novel mutation I54V in exon4 of the TR*β* gene. This mutation was found in the index case, her mother, and two asymptomatic sisters.

## 2. Case Report

A 13-year-old girl was seen in 2007 because of hypertension treated since 2005 by Acebutolol 400 mg/day and Captopril 50 mg/day. She had a full-term birth, followed by a normal development during the neonatal period. When first seen at our clinic, she weighed 67 kg (>97 percentile), her height was 174 cm (+3 SD), her blood pressure was 150/10 mmHg, and her pulse rate was 90 beats/min. Her thyroid gland was normal but she had clinical symptoms of hyperthyroidism (finger tremor and moist skin) but no ocular signs or symptoms. She had no family history of thyroid diseases. At that time, her thyroid function tests revealed free T4, 17.9 than 25 pmol/L (normal range 10.3–24.4), and TSH, 7.84 than 5.68 *μ*UI/mL (normal range 0.4–4.0). These tests were controlled by radioimmunological assay, and the investigations showed free T4, 15.2 pmol/L (normal range, 11–25), free T3, 9.5 pmol/L (normal range, 3.9–6.8), and TSH, 18.5 *μ*UI/mL. (normal range, 0.17–4.0). All the other causes of secondary hypertension were eliminated (renal causes, Cushing's syndrome, acromegaly, pheochromocytoma, and hypermineralocorticism). Anti-TPO antibodies (238 UI/mL) were positive suggesting mild chronic thyroiditis. Ultrasonography showed no goiter but a heterogeneous thyroid. All family members had normal FT4 and TSH levels (Table 1). 

**Figure 1:**
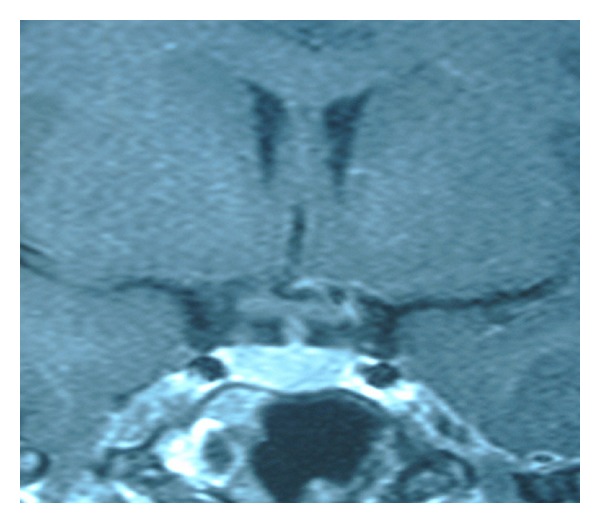
Magnetic resonance imaging of the sellar region of our patient: no abnormal findings.

**Figure 2:**
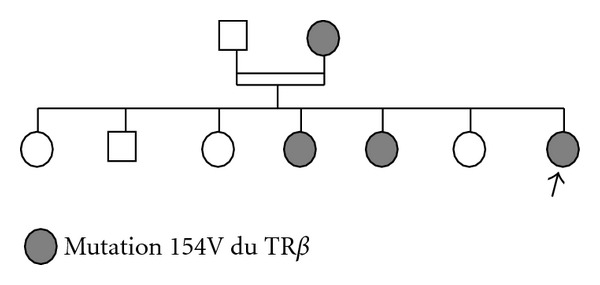
Pedigree of the family.

The level of TSH *α*-subunit was 0.33 *μ*IU/mL (range, 0.05–0.9) and the ratio TSH *α*-subunit/TSH was 1. Magnetic resonance imaging of the sellar region showed no abnormal findings (Figure 1). Sequence analysis of the TR*β* gene was performed in the index case, her both parents, her five sisters, and her four nephews. All members gave their informed consent to participate in this study. Leukocyte DNA was extracted from blood samples using standard methods. The result revealed a novel mutation I54V in exon4 of the TR*β* gene with a substitution of isoleucine for valine, and the patient was diagnosed as having a case of THR syndrome. The same mutation was also found in the mother and two asymptomatic sisters (Figure 2).

Propranolol (20 mg bid) and Enalapril (20 mg/day) were given to our patient, and her hypertension and hyperthyroidism symptoms were controlled. 

Periodic thyroid function tests during followup showed FT4 at the upper limit with unsuppressed TSH until September 2009. In January 2010, the patient presented with symptoms of hypothyroidism: weight gain and slow mentation, and presence of peripheral hypothyroidism: FT4 at 4.31 pmol/L and TSH at 242.4 *μ*UI/mL (Table 2). Our patient presents therefore a rare association of THR with chronic thyroiditis. Treated with levothyroxine (100 *μ*g/day), her last thyroid function tests (May 2010) were normal: FT4, 15.57 pmol/L, and TSH, 2.34 *μ*UI/mL.

## 3. Discussion

Our index case had high levels of FT3, FT4 at the upper limit, inappropriate lack of TSH suppression, and clinical symptoms of hyperthyroidism. The TSH *α*-subunit/TSH ratio was 1, and the MRI showed no pituitary adenoma. These findings exclude the possibility of TSH-producing pituitary adenoma and suggest the presence of RTH.

RTH is found in about 1 case per 40,000 live births [10]. Familial occurrence of RTH has been documented in approximately 75% of cases [2]. Inheritance is usually autosomal dominant [2], and transmission was clearly recessive in only one family [1].

RTH is classified into two phenotypes: GRTH and PRTH. Patients with GRTH are typically euthyroid or hypothyroid, whereas patients with PRTH (such as our patient) are usually hypermetabolic [11]. No differences in the absolute levels of TSH or free thyroid hormone are observed in GRTH patients as opposed to PRTH patients. A molecular mechanism to explain these two clinical phenotypes has proven elusive, and many authors have concluded that they are part of a spectrum of the same disorder [4, 5].

Thyroid hormone receptors (TRs) are ligand-dependent transcription factors, which mediate the biological activities of T3. TRs are encoded for by the THRA and THRB genes, which are located on chromosomes 3 and 17, respectively [12]. Although THRA1 and THRB1 are ubiquitously expressed, THRA1 is expressed primarily in the heart, bone, and brain, whereas THRB1 is more abundant in the liver, kidney, and thyroid. THRB2 expression is limited to the pituitary, hypothalamus, retina, and inner ear, and THRB3 expression has been detected principally in the heart and kidney [10, 13]. 

Most cases of RTH are caused by mutations in the THRB gene. However, RTH without a structural THRB defect occurs in approximately 10% of the cases [14]. Since the first demonstration of non-TR RTH [15], 29 subjects belonging to 23 different families have been identified [2, 16–18].

 It has been postulated that a cofactor interacting with THR may be responsible for the manifestation of RTH [14]. Subjects with the same mutation may exhibit different phenotypes like our patient and her two sisters, suggesting modulation of thyroid hormone actions by other factors.

Patients who present with apparent selective pituitary resistance are the most difficult to manage. If they exhibit hyperthyroid features at tissue levels (like our patient), they generally require treatment to reduce the elevated thyroid hormone levels. 3,5,3′-Triiodothyroacetic acid (TRIAC), a physiological metabolite of T3, can reduce TSH and endogenous thyroid hormone levels and alleviate symptoms [19]. However, the efficacy of TRIAC is variable [20] and its effect on heart rate is often minimal, probably because the decrease in thyroid hormone levels is offset by the intrinsic thyromimetic effect of the drug. Our patient did not receive TRIAC, and her symptoms of hyperthyroidism improved after treatment with propranolol. 

Concomitant autoimmune thyroiditis in patients with RTH is rarely reported [9, 21, 22]. As our patient was positive for anti-TPO antibodies, we diagnosed her as having RTH with coincidental autoimmune thyroiditis. This thyroiditis may explain the lack of important elevation of FT4. The hypothyroidism that she developed in 2010 is due to aggravation of the chronic thyroiditis and required treatment with thyroid hormone.

In conclusion, we have described the 3-year history of a young girl with RTH and chronic thyroiditis. She showed a particular phenotype that included clinical hyperthyroidism, hypertension, and accelerated growth, without goiter or ocular signs. This clinical presentation is not habitual in RTH because growth retardation is frequently reported in this syndrome. The association between RTH and thyroiditis complicates the management of the hypothyroidism because the TSH concentration cannot be a reliable marker in monitoring replacement therapy with thyroid hormone.

Our patient has a novel mutation I54V in the TRB gene. Her mother and her two sisters were asymptomatic although they have the same mutation, and they need a long-term followup for the eventual appearance of an abnormal thyroid function.

## Figures and Tables

**Table 1:** Biochemical findings of the family members.

	FT4 (pmol/L)	TSH (*μ*UI/mL)
The index patient	See the text and Table 2	
Father	13.1	1.03
Mother	17.6	3.86
1st sister	17.8	1.53
2nd sister	17.8	2.31
3rd sister	17.4	1.55
4th sister	18.3	1.49
5th sister	20.2	1.9
Normal range	10.3–24.4	0.4–4.0

**Table 2:** Evolution of the biochemical findings of the index patient.

	3/7/07	6/7/07	9/7/07	4/1/10	18/1/10	5/5/10
	Laboratory 1	Laboratory 1	Laboratory 2	Laboratory 1		
	(Immunochemiluminescence)	(Immunochemiluminescence)	(radioimmunology)	(Immunochemiluminescence)		
FT4 (pmol/L)	17.9	25	15.2	4.31	5.79	15.57
FT4 (normal range)	10.3–24.4		11–25	10.3–24.4		
TSH (*μ*UI/mL)	7.84	5.68	18.5	242.4	197	2.34
TSH (normal range)	0.4–4.0		0.17–4.0	0.4–4.0		
FT3 (pmol/L)	—	—	9.5	—	—	—
FT3 (normal range)	—		3.9 – 6.8	—		
